# Dose-dependent LDL-cholesterol lowering effect by plant stanol ester consumption: clinical evidence

**DOI:** 10.1186/1476-511X-11-140

**Published:** 2012-10-22

**Authors:** Kirsi Laitinen, Helena Gylling

**Affiliations:** 1 Benecol Division, Raisio Group, Raisio, Finland; 2 Department of Medicine, Division of Internal Medicine, University of Helsinki, Helsinki, Finland; 3 Department of Clinical Nutrition, Institute of Public Health and Clinical Nutrition, University of Eastern Finland, Kuopio, Finland

**Keywords:** Plant stanols, Plant stanol ester, Phytosterol, LDL cholesterol, Dose–response

## Abstract

Elevated serum lipids are linked to cardiovascular diseases calling for effective therapeutic means to reduce particularly LDL-cholesterol (LDL-C) levels. Plant stanols reduce levels of LDL-C by partly blocking cholesterol absorption. Accordingly the consumption of foods with added plant stanols, typically esterified with vegetable oil fatty acids in commercial food products, are recommended for lowering serum cholesterol levels. A daily intake of 1.5 to 2.4 g of plant stanols has been scientifically evaluated to lower LDL-C by 7 to 10% in different populations, ages and with different diseases. Based on earlier studies, a general understanding is that no further reduction may be achieved in intakes in excess of approximately 2.5 g/day. Recent studies however suggest that plant stanols show a continuous dose–response effect in serum LDL-C lowering. This review discusses the evidence for a dose-effect relationship between plant stanol ester consumption and reduction of LDL-C concentrations with daily intakes of plant stanols of 4 g/day or more. We identified five such studies and the overall data demonstrate a linear dose-effect relationship with the most pertinent LDL-Cholesterol lowering outcome, 18%, achieved by a daily intake of 9 to 10 g of plant stanols. Along with reduction in LDL-C, the studies demonstrated a decrease in cholesterol absorption markers, the serum plant sterol to cholesterol ratios, by increasing the dose of plant stanol intake. None of the studies with daily intakes up to 10 g of plant stanols reported adverse clinical or biochemical effects from plant stanols. In a like manner, the magnitude of decrease in serum antioxidant vitamins was not related to the dose of plant stanols consumed and the differences between plant stanol ester consumers and controls were minor and insignificant or nonexisting. Consumption of plant stanols in high doses is feasible as a range of food products are commercially available for consumption including spreads and yoghurt type drinks. In conclusion, a dose-effect relationship of plant stanols in higher doses than currently recommended has been demonstrated by recent clinical studies and a meta-analysis. Further studies are called for to provide confirmatory evidence amenable for new health claim applications and dietary recommendations.

## Introduction

Reduction of serum cholesterol and particularly LDL-cholesterol (LDL-C) is important because high levels are associated with an increased risk of atherosclerosis and coronary heart disease [1,2]. Preventive life-style related means to reduce serum cholesterol are being actively searched for to reduce the burden of cardiovascular disease on population levels [3]. Plant stanols and sterols (phytosterols) are a group of compounds that are found in plant-based foods like cereals and vegetable oils. Structurally they resemble cholesterol, but they exert much lower absorption rates compared to cholesterol. Plant stanols and sterols have been shown to reduce serum levels of cholesterol by partly blocking the cholesterol absorption in the digestive tract [4].

Plant sterols and stanols are typically esterified to vegetable oil fatty acids to ease the formulation of food applications and to ensure the cholesterol lowering effect in a wide range of food products. Plant stanols are saturated forms of plant sterols. Currently there are two main phytosterol-based ingredients used in commercial foods; plant stanol ester that is based almost exclusively on plant stanols, i.e. sitostanol and campestanol and plant sterol ester, that is mainly based on plant sterols (sitosterol and campesterol).

Indeed, it has been shown in several randomized, double blinded clinical studies that consumption of foods like margarine or yoghurt type drink with added plant stanol ester effectively reduce serum total cholesterol and LDL-C. The Scientific Panel on Dietetic Products, Nutrition and Allergies to the European Commission stated in its evaluation report: “On the basis of the data presented, a clinically significant LDL-C lowering effect of about 10% can be achieved by a daily intake of plant stanol esters equivalent to 2 g of plant stanols in an appropriate food (e.g. fat-based foods and low-fat foods such as yoghurt), preferably with meals”. The Panel considers that such a reduction is of biological significance in terms of reduced risk of coronary heart disease [5]. Meta-analyses, in which either no or only a few large-dose studies were available or in which the plant sterol and stanol studies were analysed together, have arrived at similar conclusions that no added benefit is achievable by consuming phytosterols (i.e. plant sterols or plant stanols) in excess of about 2,5 g / day [6-8]. However, Musa-Veloso and co-workers [9] separately studied the dose-range effect of plant sterols and stanols on serum LDL-C lowering and reported that plant stanols showed a continuous dose–response in serum LDL-C lowering, whereas no further serum LDL-C was apparent with plant sterols at daily intakes exceeding 2 g/day.

Recently published studies conducted with larger doses of plants stanols suggest that a further benefit to blood lipid values may be achieved by higher daily intakes of plant stanols. The aim of the present review is to discuss the evidence for a dose-effect relationship between plant stanol ester consumption and reduced serum LDL-C concentrations with daily intake exceeding the currently recommended 2 g per day of plant stanols.

### A dose response effect between plant stanol consumption and serum LDL-C concentration

The scientific evidence for the cholesterol and LDL-C lowering effect of plant stanol ester consumption in amounts 2 to 3 g plant stanols per day is sound and is supported by numerous human clinical intervention trials. There is increasing evidence that higher daily intakes may result in a greater serum LDL-C lowering effect and that the dose–response relation is linear over a wider range of intake than earlier recognized. We are now examining the studies in which plant stanols have been consumed in doses of 4 g or more. We could identify five such studies from literature search [10-14]. One of the studies has been presented only as a conference-proceedings with limited available data on study conductance [10].

An overview of the study designs and subject characteristics is presented in Table 1. Four of the studies were of high scientific quality as the studies were placebo-controlled, randomized studies, one with cross-over design [12] and three with parallel design [11,13,14]. One study investigated the dose–response effect of consuming plant stanols in an open label study with three study groups where the comparison of the dose–response effect was conducted with respect to the baseline levels, but no control group was included in the study [10]. One of the studies enrolled only eight subjects, but the study was nevertheless of sufficient statistical power to demonstrate an LDL-C lowering effect by plant stanols [12]. The interventions lasted from 2 to 10 weeks, which is a sufficient time period to induce the plant stanol ester specific blood cholesterol lowering effect [15]. The studies were conducted with mildly hypercholesterolaemic, slightly overweight adults, except one study which was conducted with normal weight, normocholesterolaemic adults.

**Table 1:** Characteristics of studies investigating LDL-cholesterol lowering effect of plant stanols consumption in doses of 4 g or more

**Reference**	**Subject characteristics**					**Treatment characteristics**			
	N	Age (years)	Men (%)	weight status	Baseline bloodcholesterol	Study design	Vehicle	Dose of plant stanols (g/d)^1^	Duration (wk)
[10]Nguyen 1999	83	49	30		mildly hypercholesterolaemic	open label, dose-response in comparison to respective baselines	margarine, soya yoghurt	3 / 6 / 10	2
[11]Plat & Mensink 2000	112	33	37	normal	normocholesterolaemic	parallel, randomized, double-blind, placebo-controlled	margarine, shortening for baking	0 / 3.8^2^ / 4^3^	8
[12]Cater et al. 2005	8	58	75	slightly overweight	mildly hypercholesterolaemic	cross-over,randomized, double-blind, placebo-controlled	margarine	0 / 2 / 3 / 4	6
[13]Gylling et al. 2010	49	62	35	slightly overweight	mildly hypercholesterolaemic	parallel, randomized, double-blind, placebo-controlled	margarine, oat based drink	0 / 8.8	10
[14]Mensink et al. 2010	93	56	53	slightly overweight	mildly hypercholesterolaemic	parallel, randomized, double-blind, placebo-controlled	margarine, soya based yoghurt	0 / 3 / 6 / 9	4

^1^ in each study group, ^2^vegetable oil based plant stanols, ^3^ pine wood based plant stanol.

**Figure 1:**
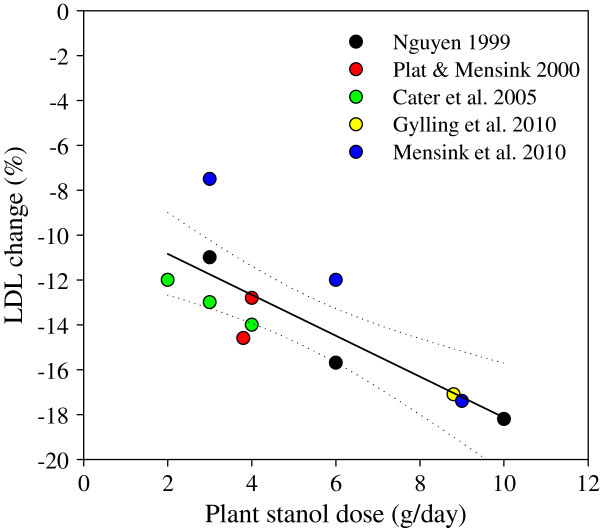
**Dose**-**effect relationship between plant stanol dose and LDL-C change (%) in five studies with plant stanol doses between 2 to 10 g/day.** Regression line (continuous line) and 95% confidence interval (dotted lines) were calculated using the least-squares linear regression. r^2^=0.66, p=0.001.

**Figure 2:**
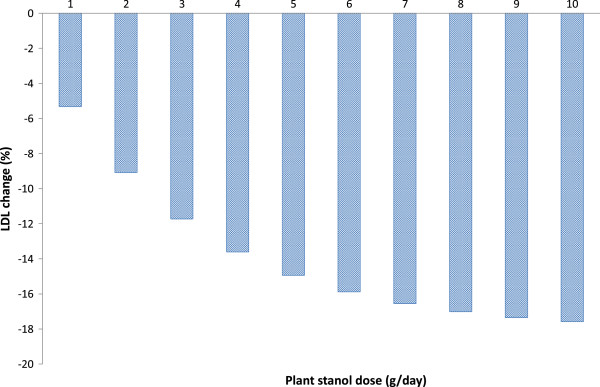
Predicted dose-effect relationship between plant stanol dose (g/day) and LDL-C change (%) estimated according to equation (predicted relative change in LDL-cholesterol, weighted analysis, no dose restriction) by Musa-Veloso et al. 2011.

The effect of plant stanol ester consumption in lowering LDL-C concentration in the five studies is presented in Figure 1. The dose–response relationship between plant stanol dose (g/day) and LDL-C change (%) was tested using the least-squares linear regression. All studies and doses were equally weighted. The dose–response is described by the following equation: LDL-C change (%) = −9.02 – 0.91 × dose (r2=0.66, p=0.001). The dose–response effect is shown by a higher proportional decrease in LDL-C concentration as a response to an increasing dose of plant stanol consumption. The most prominent effect in LDL-C lowering was demonstrated by doses 9 to 10 g of plant stanols. The highest doses of plant stanol esters resulted in a reduction of LDL-C by about 18%. A previous meta-analysis by Musa-Veloso and co-workers [9] with partially the same studies, demonstrated an LDL-C lowering efficacy by plant stanols over a continuous dose range from 0.45 g to 9 g/day. From this report, the predicted dose–response relationship between plant stanol dose (g/day) and LDL-C change (%), estimated according to equation for predicted relative change in LDL-C with weighted analysis and no dose restriction, is depicted in Figure 2.

### Scientific support for a dose-dependent serum LDL-C lowering with plant stanol ester

Plant stanols reduce the absorption of both cholesterol and plant sterols such as sitosterol and campesterol in the gastrointestinal tract with a subsequent serum cholesterol and plant sterol lowering effect. Serum levels of plant sterols is a validated marker of cholesterol absorption efficiency [16], although some caution has been raised against the validity of this marker under all circumstances [17]. In clinical studies with plant stanol ester, the reduction in cholesterol absorption can be evaluated by measuring relative changes in serum plant sterols from the baseline to the end of the plant stanol ester intervention. Miettinen and co-workers [18] showed that the higher the daily consumption of sitostanol, the bigger the reduction in serum cholesterol. The research group also recorded a significant association between the reduction in serum cholesterol levels and changes in serum campesterol concentrations due to sitostanol ester consumption. A similar association between the reduction in serum/plasma campesterol and sitostanol (3 g/day) intake was recorded a couple of years later by Gylling et al. [19]. The recorded 15% reduction in serum LDL-C in the intervention group versus the control group resulted in 44.5% reduction in fractional cholesterol absorption from the gastrointestinal tract. Considering serum plant sterol levels, the measured relative reduction in serum campesterol and sitosterol levels were 41.7% and 29.6%, respectively.

Three of the studies with high daily plant stanol intake also reported serum plant stanol and sterol concentrations. Gylling and co-workers [13] demonstrated that a daily intake of 8.8 g plant stanols decreased cholesterol absorption markers (serum plant sterol to cholesterol ratios) by up to 62%, the mean proportional change from the baseline being 44.9 for campesterol and 46.2 for sitosterol. Similarly, Mensink and co-workers [14] showed continuous decrease in plasma sitosterol and campesterol concentrations with 3, 6 and 9 g intakes of plant stanols in comparison to controls, although the difference in changes for the 6-g and 9-g groups were not statistically significantly different from the previous dose. With increasing plant stanol intake, the cholesterol absorption was reduced [14]. Nguyen [10] reported a 40% lower campesterol and 50% lower sitosterol levels after daily consumption of 10 g of plant stanols.

The decrease in the LDL-C level by 18% due to consumption of plant stanols in high doses (about 9 g) is of similar magnitude to that achieved by drugs lowering cholesterol via partly blocking cholesterol absorption. Ezetimibe is a drug that reduces the uptake of both cholesterol and plant sterols by its action on the Nieman-Pick C1-Like1 (NPC1L1) transporter protein. A daily dose of 10 mg ezetimibe reduces serum LDL-C levels by some 17 – 22% versus controls [20]. Sudhop and co-workers [21] recorded a 20.4% reduction in serum LDL-C from baseline with ezetimibe 10 mg/day. Fractional cholesterol absorption was reduced by 54.4% and circulating levels of campesterol and sitosterol were reduced by 48% and 41% respectively.

Thus, the higher relative reduction in serum plant sterol levels obtained with higher daily intake of plant stanols supports the notion that a higher daily intake of plant stanols results in enhanced inhibition of cholesterol absorption and further serum LDL-C reduction with increasing daily intakes of plant stanols.

### Consumption of plant stanol esters in doses of up to 10 g per day is safe and feasible

Plant stanols are generally considered safe as they are absorbed within the gastrointestinal tract only in minuscule quantities [22]. None of the clinical studies with daily intakes up to 10 g of plant stanols reported any side effects or changes in clinical characteristics like blood pressure or indicators of liver or kidney function or blood coagulation [11,13,14].

The 0.04 to 0.2% of the consumed plant stanol dose is absorbed from the gastrointestinal tract. Subsequently the circulating sitostanol concentration is raised from the typical natural concentrations of 10–15 μg/dl to 20–30 μg/dl. At higher intakes of 8.8 g/day of plant stanols, the serum sitostanol levels have been demonstrated to double from baseline level of 17 ug/dl and campesterol levels to increase from non-detectable levels to 9 ug/dl but to remain at a comparable level as with the lower intake of 2 to 3 g/day [[13]]. Further, the levels normalize in four weeks after cessation of the consumption [13]. In the study by Mensink and co-workers [14] a gradual increase for plasma plant stanols was detected with increasing plant stanol consumption from 3 to 6 to 9 g/day, but again the effects were leveling off at higher intakes. Thus whilst reducing effectively serum cholesterol and plant sterol levels, high intakes of plant stanols do not appear to increase the systemic availability of plant stanols more than lower doses and appear not to influence cognitive function [[23]].

A concern over plant stanol consumption has been directed at a potential decrease in the serum concentrations of lipid-soluble antioxidants and vitamins. Such decreases in the concentrations of serum β-carotene, α-tocopherol and lutein [14] and also α-carotene [13] by a few percent have indeed been detected in some studies. The observed phenomena is partly due to a reduction in circulating lipoprotein particles, the carriers of antioxidants, and when the anti-oxidant concentrations are standardized for total or LDL-C, the differences between controls and those consuming plant stanols are minor or non-existing [14]. Furthermore, the serum concentrations of vitamins, although having decreased somewhat, have been within normal reference limits. In the high-dose study by Gylling and co-workers [13] the reduction in serum β-carotene obtained with a 9 g daily intake of plant stanols was similar to that recorded with a 2–3 g daily intake of plant stanols, also carried out on a Finnish population [24]. Similarly, Nguyen [10] demonstrated that after 2-weeks of daily consumption of 10 g of plant stanol, no decrease in serum β-carotene levels was detected. No alteration in serum vitamin A [13] and vitamin D [10,13] concentrations have been detected by consumption of high doses of plant stanols. Further, dietary intake of fruits and vegetables, the sources of carotenoids, in accordance with the recommended reference values have been demonstrated to sustain serum carotenoid levels regardless of plant stanol consumption [25]. Plant stanol ester consumption does not seem to influence serum vitamin K levels [[26]] or blood coagulation and fibrinolysis [27,11]. It has been demonstrated that consumption of 4 g of plant stanols as ester had no influence on clotting factor VII or on fibrinolytic parameter, plasminogen activator antigen response, but a tendency for increase in the activity of antithrombin-111, a potent inhibitor of coagulation, was detected [11]. It should also be considered that plasma levels of vitamin K reflect very recent daily intake. No clinical study with plant stanol ester has been conducted where the daily intake of vitamin K has been monitored to exclude potential impact of variation in daily intake of vitamin K close to blood sampling. Nevertheless, there is an obvious need for further investigation into the functional effects of plant stanol ester consumption in high doses on blood coagulation and fibrinolysis particularly in patients receiving warfarin therapy.

Consumption of plant stanols in doses up to 10 g per day is nowadays feasible as a range of food products are available for consumption including spreads, mayonnaise, salad dressing, yoghurt drinks and bread. Due to this variability in choice of food products the compliance of the study subject in consuming the plant stanol containing food has been good. The food matrix in which plant stanols are incorporated does not appear to contribute to the cholesterol lowering effect of the plant stanols [9]. The fatty acid composition of high-fat products like spreads with added plant stanol esters is in line with dietary recommendations calling for an increase in consumption of unsaturated fatty acids along with decrease in saturated fatty acids. Consumption of such high-fat food products with added plant stanol esters has therefore an additional benefit as they replace in the diet the usual food product with potentially unfavorable fatty acid composition. Further, there are also several low-fat products like yoghurt type drinks commercially available that do not add fat to the diet.

## Conclusions

The scientific evidence for the cholesterol and LDL-C lowering effect as well as safety of daily consumption of plant stanol esters is well-documented in scientific literature. The most recent progress in the area of plant stanol research is the provision of evidence that intakes of plant stanols in higher doses than currently recommended result in an enhanced decrease in LDL-C concentrations comparable to that obtained by pharmacological means. Plant stanol intakes in daily doses of 9 to 10 g results in LDL-C lowering by up to 18%. The approach for lowering LDL-C with higher (4 to 6 g) daily intakes of plant stanols is feasible as a range of food products are commercially available for consumption. Enhanced serum LDL-C lowering by 10% likely contributes to beneficial effects in reducing the risk of cardiovascular diseases by 10% through dietary means [28]. The approach likely provides cost-effective benefit and may be seen as an amenable public health policy [29].

## Competing interests

K. Laitinen is clinical research manager at Raisio Group. H. Gylling has no competing interests.

## Authors’ contributions

KL drafted the first version of the manuscript. Both authors critically revised, read and approved the final manuscript.
